# Cardiac Complications in Immune Checkpoint Inhibition Therapy

**DOI:** 10.3389/fcvm.2019.00003

**Published:** 2019-01-23

**Authors:** Kazuko Tajiri, Masaki Ieda

**Affiliations:** Department of Cardiology, Faculty of Medicine, University of Tsukuba, Tsukuba, Japan

**Keywords:** immune checkpoint inhibitors, myocarditis, cardiotoxicity, programmed cell death protein 1, cytotoxic T-lymphocyte antigen 4, immune-related adverse events, immune checkpoint, autoimmunity

## Abstract

Immune checkpoint inhibitors (ICIs) have changed the treatment landscape of advanced cancers. Unfortunately, these agents can induce a wide spectrum of immune-related adverse events (irAEs) through activation of immune responses in non-target organs, including the heart. As the clinical use of ICI therapy increases rapidly, management of irAEs is becoming extremely important. The most commonly presented cardiac irAE is myocarditis. Histopathologically, T-cell (with a predominance of CD8^+^ cells) and macrophage infiltration in the myocardium is typically observed in ICI-associated myocarditis. Other presentations of cardiac irAEs include congestive heart failure, Takotsubo cardiomyopathy, pericardial disease, arrhythmias, and conduction disease. Although cardiac irAEs are relatively rare, they can be life-threatening. Hence, cardiologists and oncologists should be vigilant for these presentations.

## Introduction

Immune checkpoint inhibitors (ICIs), including monoclonal antibodies (mAbs) against cytotoxic T-lymphocyte-associated antigen 4 (CTLA-4), programmed cell death protein 1 (PD-1), and programmed cell death ligand 1 (PD-L1), are being routinely used in clinical settings and have shown unprecedented efficacy in treating multiple cancers (1–6). Unfortunately, these agents can induce a wide spectrum of immune-related adverse events (irAEs) (7–9) through activation of immune responses in non-target organs, including the heart. In recent years, several cases of cardiotoxicity have been reported in cancer patients treated with ICIs (10–17). Although its frequency is lower than that for other irAEs, cardiotoxicity can become life-threatening, making it an important consideration for cardiologists, oncologists, and immunologists.

In this review, we describe the mechanisms and summarize the reported clinical scenarios of cardiotoxicities associated with ICIs. Evidence available for diagnosis, management, and prognosis are considered.

## Physiological Roles of Immune Checkpoints

T lymphocytes play a pivotal role as modulators and effectors of the immune system. Naïve T cells are activated after recognizing a cognate peptide presented by antigen-presenting cells (APCs) via interaction between the major histocompatibility complex (MHC) on the APCs and the T cell receptor (TCR), but further co-stimulatory signals are required for activation. CD28 is a stimulatory co-receptor expressed on T cells. Binding of CD80 (also known as B7-1) or CD86 (also known as B7-2) molecules on APCs with CD28 molecules provides an essential signal for T cell activation. However, to prevent destructive immune activation, these signals are finely regulated by immune checkpoints (e.g., CTLA-4 and PD-1) (18).

### CTLA-4

CTLA-4 is an inhibitory co-receptor expressed on activated T cells. CTLA-4 inhibits T cell functions by competing with CD28 for binding with B7 ligands, CD80, and CD86. CTLA-4 is homologous to CD28 but has much higher binding affinity and avidity for B7 ligands. In resting naïve T cells, unlike CD28, which is constitutively expressed on the cell surface, CTLA-4 is localized primarily in intracellular vesicles (19). CTLA-4 is upregulated on the cell surface in response to TCR activation and the signal is enhanced by co-stimulation through CD28 and/or interleukin-2 (20). Of note, the stronger the TCR signal, the greater the CTLA-4 translocation to the cell surface, thereby preventing harmful T cell activation (19–21).

### PD-1:PD-L1/2 Pathway

PD-1 is another inhibitory receptor and plays a pivotal role in regulating the effector phase of T cell responses through binding with its ligands PD-L1 and programmed death ligand 2 (PD-L2) (21). PD-L1 is expressed constitutively on hematopoietic cells and a wide range of non-hematopoietic cells, including hepatocytes, astrocytes, epithelial cells, muscle cells including cardiomyocytes, vascular endothelial cells, and pancreatic cells (22, 23). PD-L1 is also expressed on numerous tumors, and its expression is reported to be associated with poor prognosis in several cancers (24–26). In contrast to PD-L1, PD-L2 is expressed primarily on APCs and certain B cell lymphomas (20, 21). Similar to CTLA-4 signaling, PD-1 signaling abrogates T cell proliferation and cytokine production and reduces T cell survival (23). PD-1 exhibits minimal expression on resting immune cells. However, upon activation, PD-1 expression is induced on the surface of T cells, B cells, natural killer cells, natural killer T cells, dendritic cells, and macrophages (23).

## Implications of Blocking the CTLA-4 and PD-1 Pathways in Cancer

**Figure 1:**
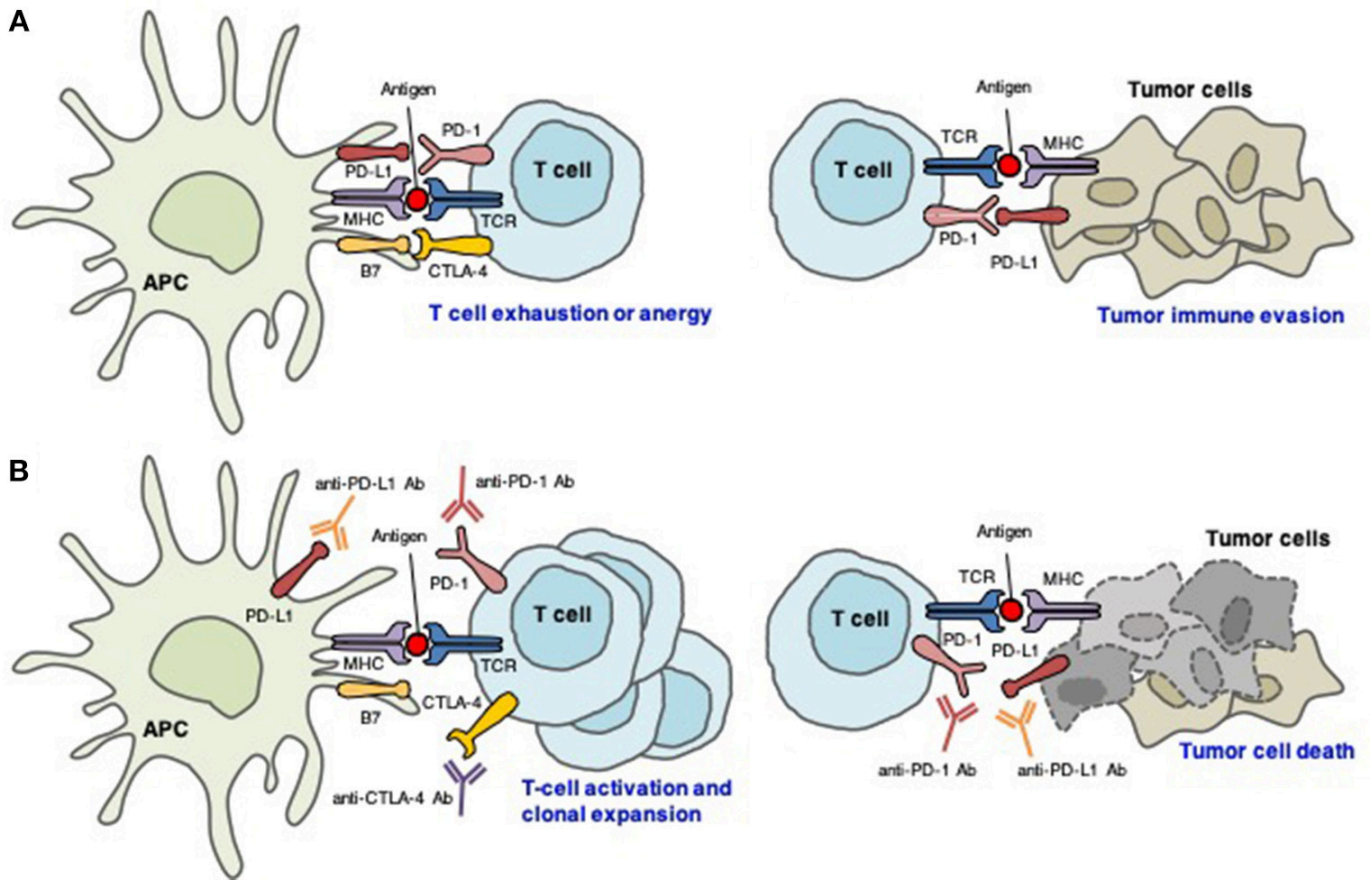
Distinct roles of CTLA-4 and PD-1/PD-L1 in the regulation of antitumor immune responses. **(A)** Interactions between CTLA-4/B7 and PD-1/PD-L1 inhibit T cell-mediated tumor cell killing. **(B)** Blockade of CTLA-4, PD-1, and PD-L1 results in T cell activation and proliferation, which reactivates T cell-mediated tumor cell killing.

To date, six ICIs have been approved by the United States Food and Drug Administration: ipilimumab (anti-CTLA-4 mAb); nivolumab and pembrolizumab (anti-PD-1 mAbs); and atezolizumab, avelumab, and durvalumab (anti-PD-L1 mAbs) (Table 1). Antibody therapies against the CTLA-4 and PD-1/PD-L1 axes have revolutionized the treatment of cancer (Figure 1).

**Table 1:** FDA-approved ICIs for cancer therapy.

**Target**	**Drug**	**Indication**
CTLA-4	Ipilimumab	Melanoma
PD-1	Nivolumab	Melanoma, NSCLC, SCLC, RCC, HCC, Hodgkin lymphoma, head and neck cancer, urothelial carcinoma, microsatellite instability-high, or mismatch repair-deficient metastatic colorectal cancer
PD-1	Pembrolizumab	Melanoma, NSCLC, head and neck squamous cell carcinoma, Hodgkin lymphoma, urothelial carcinoma, microsatellite instability-high cancer, gastric cancer, cervical cancer, primary mediastinal large B-cell lymphoma
PD-L1	Atezolizumab	NSCLC, urothelial carcinoma
PD-L1	Durvalumab	NSCLC, urothelial carcinoma
PD-L1	Avelumab	Urothelial carcinoma, Merkel cell carcinoma
CTLA-4 and PD-1 in combination	Ipilimumab and nivolumab	Melanoma, RCC, microsatellite instability-high, or mismatch repair-deficient metastatic colorectal cancer

*CTLA-4, cytotoxic T lymphocyte-associated antigen 4; FDA, Food and Drug Administration; HCC, hepatocellular carcinoma; ICI, immune checkpoint inhibitor; NSCLC, non-small cell lung cancer; PD-1, programmed cell death protein 1; PD-L1, programmed cell death ligand 1; RCC, renal cell carcinoma; SCLC, small cell lung cancer*.

Cancer is characterized by genetic mutations that can lead to the expression of various tumor-associated antigens. APCs present these antigens via MHC molecules expressed on their surface, which interact with TCRs. Thus, T cells can recognize tumor-associated antigens as “non-self” and attack tumor cells expressing these antigens (27). However, CTLA-4 inhibits T cell activation and clonal expansion. In the tumor immunotherapy setting, CTLA-4-targeting mAbs support the activation and proliferation of effector T cells, resulting in broad activation of immune responses against tumor cells (28). In contrast, CTLA-4 blockade inhibits regulatory T cell-mediated immunosuppression (28). These are thought to be the main mechanisms of action of CTLA-4 blockade.

PD-1 is expressed on tumor-infiltrating lymphocytes in many cancers. Chronic or high exposure to tumor antigens can induce persistent PD-1 expression, which leads to a state of exhaustion or anergy (lack of response). PD-1 blockade may reverse anergy of tumor-specific T cells. PD-1 is upregulated on the cell surface of many different tumor types. Tumor PD-L1 expression indicates an active tumor immune microenvironment and is strongly associated with efficacious responses to PD-1- and PD-L1-targeting mAbs (29). It is commonly accepted that PD-L1 expression on tumors and immune cells can inhibit the T-cell antitumor response and facilitate cancer development. However, the role of PD-L2 in antitumor immunity remains controversial (30, 31).

## irAEs

**Figure 2:**
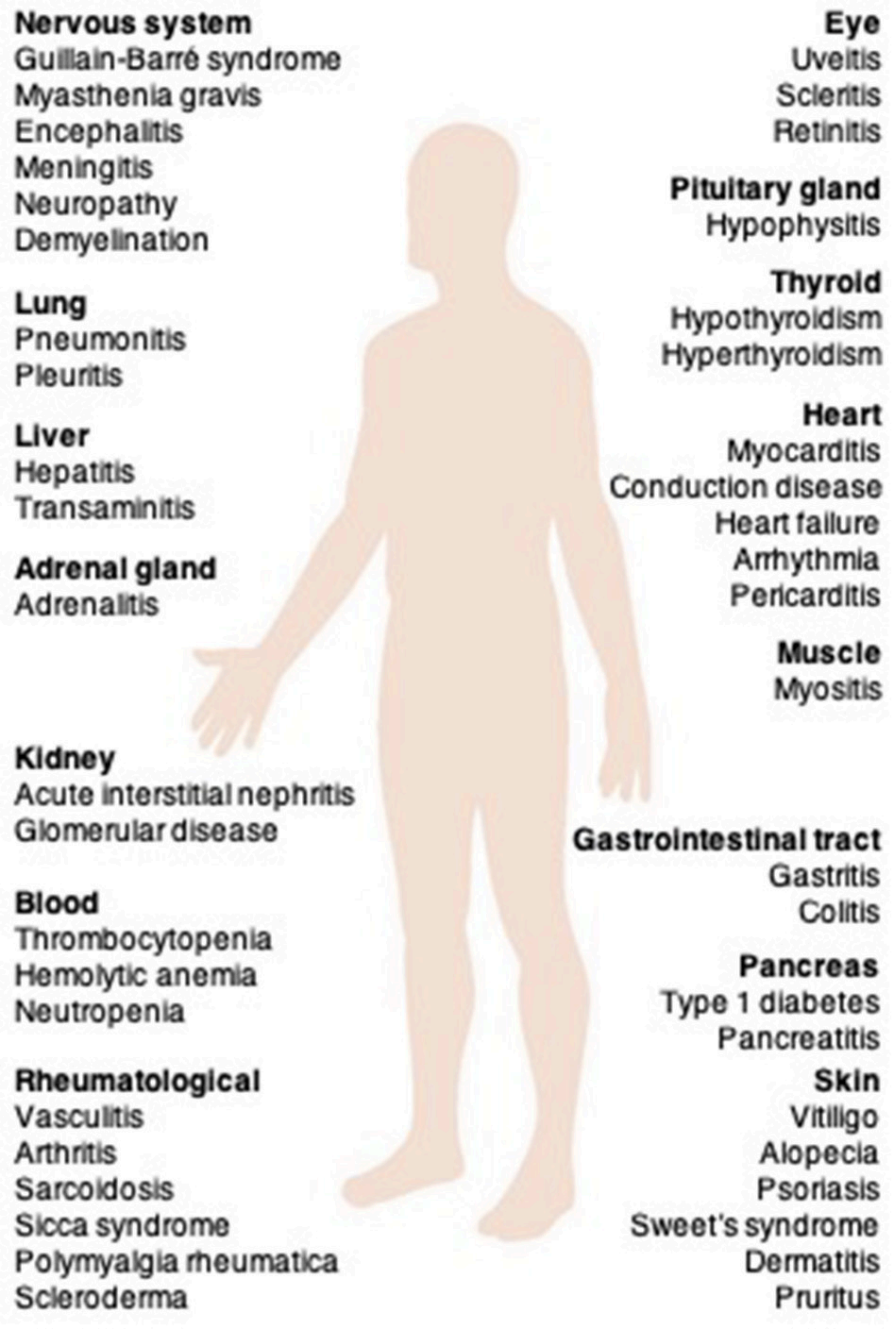
The clinical spectrum of irAEs associated with immune checkpoint inhibitors. irAEs, immune-related adverse events.

Due to the central role played by immune checkpoints in the maintenance of self-tolerance, immune checkpoint blockade can induce a spectrum of adverse events, called irAEs (32). Remarkably, irAEs can affect almost any organ system (Figure 2) and have been reported at a substantially high frequency. irAEs occur in up to 90% of patients (10–15% grade 3/4) treated with ipilimumab (1) and 79% of patients (13% grade 3/4) treated with pembrolizumab (3). In a meta-analysis, the incidence was reported to be 75% in patients treated with CTLA-4-targeting mAbs and 30% for PD-1- and/or PD-L1-blocking mAbs (33). The frequency of grade 3/4 irAEs was substantially higher in patients treated with a combination of ipilimumab and nivolumab (54%) than in those receiving ipilimumab monotherapy (20%) (34).

irAEs are generally managed with corticosteroids, and less commonly, with other immunomodulatory agents (35). There are no prospective studies that have investigated management strategies for irAEs. According to the various organs involved, grade 1–2 events mainly affect the gut and skin, whereas grade 3–4 events are mainly restricted to the digestive tract. Cardiac, neurologic, renal, ocular, and hematologic irAEs are uncommon (35).

## Cardiotoxicity Associated With Immune Checkpoint Inhibition

### Insights From Animal Studies

**Figure 3:**
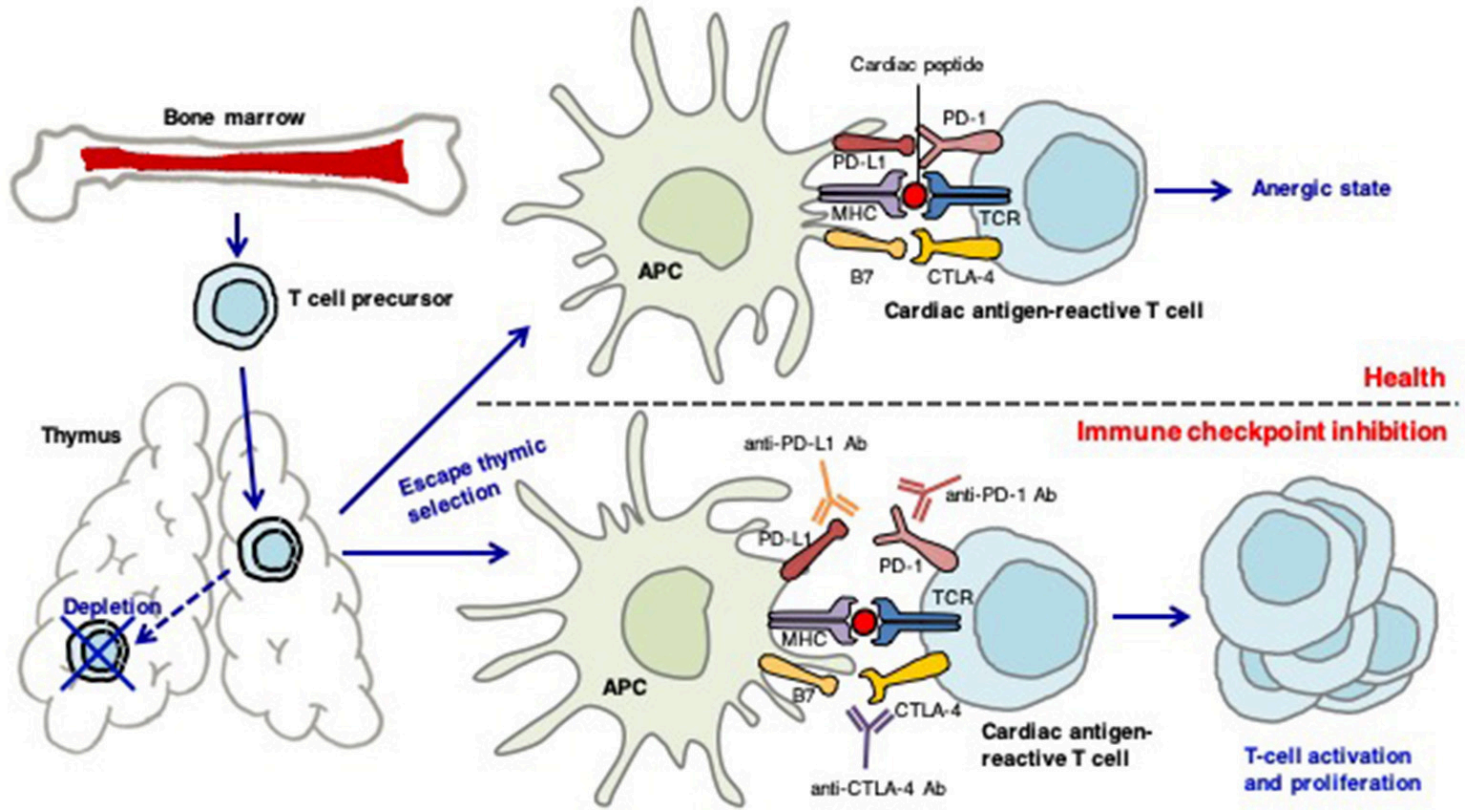
The role of immune checkpoints in establishing peripheral tolerance to the heart. During the establishment of central tolerance in the thymus, most autoreactive T cells are deleted; however, some autoreactive T cells are released into the periphery. In health, peripheral tolerance mechanisms keep these cells in check. CTLA-4 competes with CD28 to downregulate T-cell activation, resulting in immunotolerance and prevention of pathologic immune responses to cardiac-antigens. PD-1-PD-1 ligand interactions also maintain the cardiac-reactive T cells in an anergic state. Antibodies against CTLA-4, PD-1, or PD-L1 may activate cardiac antigen-reactive T cells that escape central tolerance. These T cells can clonally expand and attack the heart.

During the establishment of central tolerance in the thymus, most autoreactive T cells are deleted; however, some autoreactive T cells are released into the periphery (36, 37). In healthy individuals, peripheral tolerance mechanisms regulate the numbers of these cells. CTLA-4 competes with CD28 to downregulate T cell activation, resulting in immunotolerance and prevention of pathologic immune responses to cardiac antigens (38). Interactions between PD-1 and its ligands also maintain cardiac-reactive T cells in an anergic state (38) (Figure 3).

Mice deficient in CTLA-4 develop severe myocarditis with massive T cell infiltration (39). The cardiac presentation of PD-1-deficient mice is dependent on their background; mice on the BALB/c background develop autoimmune dilated cardiomyopathy (40), but PD-1-deficient autoimmune-prone MRL mice show lymphocytic myocarditis with massive infiltration of CD4^+^ and CD8^+^ T cells (41). PD-L1 is significantly upregulated on the surface of cardiac endothelial cells during myocarditis. PD-L1 deficiency in MRL mice induces similar severe myocarditis (42).

### Cardiac irAEs of ICIs in Patients With Cancer

#### Incidence

There are contrasting reports on the rates of cardiac irAEs associated with ICI therapies. For example, no incidence of myocarditis was identified after a pooled analysis of 448 patients treated with nivolumab and ipilimumab combination therapy (43). In contrast, a pharmacovigilance study identified myocarditis in 18 of 20,594 patients (0.09%) treated with nivolumab alone or in combination with ipilimumab (13), and a cohort study of 964 patients from a multicenter registry reported a prevalence of 1.14%, which increased to as high as 2.4% for combination therapy with anti-PD-1/anti-CTLA-4 (44). According to the latter report, ICI-associated myocarditis can no longer be considered a “rare” adverse effect. So far, ICI-associated myocarditis appears to be a class effect, and the incidence seems to be higher when patients are treated with a combination of ICIs (13, 44).

#### Clinical Presentation and Management

The most common cardiac irAE is myocarditis (45). Histopathologically, T cell (with a predominance of CD8^+^ cells) and macrophage infiltration in the myocardium are typically observed in ICI-related myocarditis (10, 12–15, 46). This myocarditis sometimes involves the cardiac conduction system, leading to conduction block. Other presentations of cardiac irAEs include congestive heart failure, Takotsubo cardiomyopathy, pericardial disease, arrhythmias, and conduction disease (47).

The time to onset of myocarditis is variable. An analysis of VigiBase, the World Health Organization's database of individual case safety reports that includes 101 cases of severe myocarditis, revealed that myocarditis was diagnosed at a median of 27 days (range, 5–155 days) after the initiation of ICI therapy, with 76% of the cases occurring in the first 6 weeks of treatment (48). A medical record review of 30 ICI-related cardiotoxicity patients from two cardio-oncology units revealed that the median onset of cardiotoxicity was 65 days (range, 2–454 days) and it occurred after a median of three (range, 1–33) infusions (49). In a multicenter cohort including 35 myocarditis patients, the median time to onset was 34 days and 81% of the patients developed cardiac irAE within 3 months (44). Notably, fatal myocarditis can develop after only a single treatment with an ICI (11, 13). Unfortunately, there is insufficient information regarding the time of onset of cardiac irAEs relating to specific treatment regimes. In general, most irAEs were reported to occur within 3–6 months of the initiation of anti-CTLA-4- or anti-PD-1-targeting therapy (47, 50). While the risk of severe irAEs appears to be dose-dependent with anti-CTLA-4 antibodies, toxicities with anti-PD-1/anti-PD-L1 antibodies are reported to be dose-independent (47, 50).

Cardiac signs and symptoms of ICI-related cardiotoxicity vary from asymptomatic to sudden death (10–17, 51) and lack specificity. Sometimes cardiac irAE is accompanied by other organ irAEs, especially those in skeletal muscles (11, 13).

Currently, patients who are likely to develop cardiac irAEs cannot be identified before ICI therapy. Therefore, early detection of ICI-related myocarditis is thought to be important for improved management. Mahmood et al. (44) showed that measuring troponin levels at baseline and at each cycle of ICI treatment may be useful for surveillance because this parameter was abnormal in 94% of ICI-myocarditis patients at clinical presentation. In contrast, Sarocchi et al. (52) measured troponin levels at each nivolumab administration in 59 patients and found an elevation in only six patients, none of whom developed overt cardiac irAEs. These researchers mentioned possible reasons for a “false positive” elevation of troponin, including it being a consequence of myocardial oxygen demand-supply mismatch due to aggravation of the clinical status or the presence of subclinical ICI-induced myocarditis. An elevation of troponin indicates the presence of, but not the underlying reason for, myocardial injury. Therefore, myocarditis or other myocardial damage should be considered in cases presenting with elevated troponin, and these patients should be referred immediately to cardiologists/onco-cardiologists for further evaluation (44). Mahmood et al. (44) also reported abnormal electrocardiograms in 89% of ICI-related myocarditis patients. In contrast, NT-ProBNP was abnormal in 66%, and the left ventricular ejection fraction (LVEF) was abnormal in only 49% of these patients. Thus, NT-proBNP or LVEF may be less useful for early diagnosis of cardiac irAEs than troponin or ECG. Information on the utility of hsCRP as an early biomarker of cardiac irAEs is lacking.

Pharmacovigilance data show that the mortality of ICI-associated myocarditis exceeded 60% in patients receiving ipilimumab-nivolumab combination therapy (13). Mahmood et al. (44) reported that nearly half of all myocarditis cases developed a major adverse cardiac event (a combination of cardiovascular death, cardiac arrest, cardiogenic shock, and hemodynamically significant complete heart block). Escudier et al. (49) reported after a medical record review of 30 ICI-related cardiotoxicity patients that eight (27%) died of cardiovascular complications. They also reported that cardiovascular mortality was significantly associated with conduction abnormalities and ipilimumab-nivolumab combination therapy (49).

Currently, there are no guidelines for the treatment of cardiac irAEs. Steroids have been used to treat cardiac irAEs in most cases (11, 13–16, 44, 45). For steroid non-responders, other immunosuppressive agents, high-dose intravenous immunoglobulin therapy, plasmapheresis, and immunoadsorption therapy have been used (45, 53).

## Conclusions

ICI therapies have changed the treatment landscape of advanced cancer. As the clinical use of ICI therapy rapidly increases, management of irAEs is extremely important. Cardiac irAEs are relatively rare but can be life-threatening. Large scale, prospective, and longitudinal cohort studies are needed to clarify predisposing risk factors and long-term consequences of ICI-induced cardiac irAEs. In addition to clinical studies, basic studies are crucially needed to provide insights into underlying mechanisms and to find biomarkers to identify high risk patients and minimize the risk of ICI-associated cardiac irAEs.

## Author Contributions

KT has written the main manuscript text and prepared the figures. MI participated to the redaction of the manuscript and its extensive revision.

### Conflict of Interest Statement

The authors declare that the research was conducted in the absence of any commercial or financial relationships that could be construed as a potential conflict of interest.

## Abbreviations

α-MHC: myosin heavy chain α isoform
APC: antigen-presenting cells
CTLA-4: cytotoxic T-lymphocyte-associated antigen 4
ICI: immune checkpoint inhibitor
irAE: immune-related adverse event
mAb: monoclonal antibody
PD-1: programmed cell death protein 1
PD-L1: programmed cell death ligand 1
TCR: T cell receptor.
